# *Mycoplasma gallisepticum* triggers immune damage in the chicken thymus by activating the TLR-2/MyD88/NF-κB signaling pathway and NLRP3 inflammasome

**DOI:** 10.1186/s13567-020-00777-x

**Published:** 2020-04-10

**Authors:** Chunli Chen, Jichang Li, Wei Zhang, Syed Waqas Ali Shah, Muhammad Ishfaq

**Affiliations:** 1 Heilongjiang Key Laboratory for Animal Disease Control and Pharmaceutical Development, College of Veterinary Medicine, Northeast Agricultural University, 600 Changjiang Road, Xiangfang District, Harbin, 150030 China; 2 College of Animal Science and Technology, Northeast Agricultural University, Harbin, China

## Abstract

Previous studies reported that *Mycoplasma gallisepticum* (MG) causes immune dysregulation in chickens. However, the underlying mechanisms of immune dysregulation in chickens are still unclear. The thymus is a primary lymphoid organ where the proliferation, differentiation and selection of T-lymphocytes occur, whereas T-lymphocytes play a crucial role in innate immune responses. To evaluate the effects of MG-infection on chicken thymus, White Leghorn chickens were divided into (1) control group and (2) MG-infection group. ATPase activities were detected by commercial kits. The hallmarks of inflammation, autophagy and energy metabolism were examined in chicken thymus tissues by histopathology, transmission electron microscopy, immunofluorescence microscopy, RT-PCR and western blotting. Immunofluorescence examination revealed that the number of CD8^+^ lymphocytes has significantly reduced in MG-infection group. In addition, morphological analysis revealed that MG induced inflammatory cells infiltration. The mitochondria were swollen and chromatin material was condensed in MG-infection group. The mRNA and protein expression results showed that MG-infection triggered the nucleotide-binding oligomerization domain, leucine rich repeat and pyrin domain containing 3 (NLRP3) inflammasome through TLR-2/MyD88/NF-κB signaling pathway. Meanwhile, the expressions of autophagy-related genes were reduced both at mRNA and protein level in MG-infection group. While, ATPase activities and the expression of energy metabolism-related genes were reduced in the thymus of MG-infected chickens. These results showed that MG-infection triggered inflammatory response through TLR-2/MyD88/NF-κB signaling pathway, activated NLRP3 inflammasome, reduced the level of autophagy and impaired energy metabolism, which then lead to tissue damage in chicken thymus. The data provide new insights in MG-infection-mediated immune damage and provide possible therapeutic targets for future targeted therapy.

## Introduction

*Mycoplasma gallisepticum* (MG) causes severe inflammation and primarily infects trachea, lungs and air sacs in chickens [1]. Previous reports demonstrated that MG is an extracellular pathogen with a total lack of bacterial cell wall and has the ability to adhere and colonize in mucosal surface epithelium [2–4], resulting in inflammatory signs like coughing, tracheal rales and sneezing [5, 6]. MG caused worldwide economic losses to chicken farming due to downgrading of carcasses, decreased feed conversion efficiency, and reduced hatchability and egg production [6, 7]. Recently, researchers demonstrated that MG induced a profound immune dysregulation and setting the stage for disease manifestations in chickens’ tracheal mucosa [8]. However, the exact mechanism of MG-infection-mediated immune dysregulation is still elusive, which play a crucial role in the pathogenesis of MG-infection.

The thymus is a central and primary lymphoid organ, where development, differentiation, maturation and selection of T-lymphocytes is orchestrated [9]. In general, thymic injury can cause serious consequences to immune development and immature immune system [10]. Accumulative evidence showed that multiple pathogens can target the thymus in mammals, resulting in functional disorder and organ atrophy [11, 12]. In birds, pathogens including viruses, bacteria and parasites were reported to cause thymic atrophy [13]. The development and recruitment of T-lymphocyte is a complex process, for instance, double-positive thymocytes passed through a series of culling process involving programmed cell death that results in terminally differentiated CD8^+^ or CD4^+^ single positive cells [14]. Previous studies reported that thymus injury was commonly found during infections [11, 15], which is indirectly related to immune impairment. However, studies are needed to elucidate the effect of MG-infection on thymus function in chickens.

Inflammasomes are cytosolic molecular sensors which belong to Nod-like receptor (NLR) family [16]. Studies demonstrated that aberrant inflammasome activation causes a variety of immune disorders [17]. Among NLR’s, nucleotide-binding oligomerization domain, leucine rich repeat and pyrin domain containing 3 (NLRP3) is one of the most studied NLR. NLRP3 inflammasome assembly is activated by a variety of signals such as reactive oxygen species (ROS), pathogen-associated molecular patterns (PAMPs), and/or damage-associated molecular patterns (DAMPs) [18]. Although inflammasome activation has not yet been reported in MG-infection in chicken thymus, the activation of NLRP3 inflammasome has been reported for other mycoplasmal species such as *Mycoplasma hyorhinis*, *Mycoplasma pneumonia* and *Mycoplasma salivarium* [19]. However, further studies are needed to understand the crosstalk between inflammasome and autophagy during bacterial infections. Autophagy is a versatile homeostatic pathway and ubiquitous in host defense against a number of microbes [20, 21]. Earlier reports showed that autophagy is at the crossroad of multiple homeostatic pathways that control inflammation and kill pathogens [22]. Our previous studies reported that MG induced autophagy in RAW264.7 cells through extracellular regulated protein kinase (ERK) signaling pathway [23]. Autophagy removes pathogens or damaged organelles, resulting in the decrease of DAMPs or PAMPs, and thus indirectly limits the stimulation/activation of inflammasomes [18]. Contrastingly, loss or inhibition of autophagy by pharmacological compounds causes the activation of inflammasomes and their downstream signaling pathways, induces mitochondrial damage, increases DAMPs or PAMPs levels [24], and leads to impairment in energy metabolism system [25]. Our previous results showed that MG-infection caused structural damage and induced oxidative stress and apoptosis in chicken thymus [26]. However, the mechanism of inflammasome activation in connection with autophagy is still not reported in detail, which could be involved in immune damage in chicken thymus. Therefore, the objectives of the present study were to investigate the molecular mechanism of MG-mediated immune damage in chicken thymus. We examined TLR-2/MyD88/NF-κB signaling pathway and NLRP3 inflammasome activation in the context of MG-infection in chicken thymus that could be possibly associated with immune damage and strongly correlated with inflammatory responses. The data showed that MG-infection inhibited autophagy and caused energy metabolism dysfunction in chicken thymus. The study provided a better understanding of the mechanism of immune dysregulation in chicken thymus and exploited new therapeutic targets for the prevention of MG-infection.

## Materials and methods

### Culture of bacteria

MG R_low_ strain was obtained from Veterinary Research Institute, Chinese Academy of Agricultural Sciences (Harbin, China). MG were grown in a modified Hayflicks medium as mentioned in our earlier study [27]. In brief, 10% freshly prepared yeast extract, 0.1% Nicotinamide adenine dinucleotide (NAD), 0.05% thallium acetate, 0.05% Penicillins and 20% fetal bovine serum (FBS) were added in modified Hayflicks medium. Chickens were challenged at a density of 1 × 10^9^ (color change unit per milliliter (CCU/mL)).

### Chickens and treatments

One-day-old specific-pathogen-free (SPF) White Leghorn chickens were purchased from Chia Chau chicken farm (Heilongjiang, China). Chickens were reared for 4 weeks and acclimatize to experimental conditions, and divided into (1) control group and (2) MG-infection group. The chickens in control group were not given mock infection. Each group is assigned 30 chickens, fresh drinking water and feed were provided ad libitum throughout the experiments. Chickens were infected with MG strain R_low_ (1 × 10^9^ CCU/mL) in the bilateral air sac as mentioned previously [28].

### Samples collection

Chickens were humanely sacrificed at day 1, day 3 and day 7 post-infection. Thymus samples were collected, washed in cold phosphate buffer saline (PBS) solution and divided into two parts. One part was used for ultrastructural, histological examination and ATPase activities. The other part was immediately stored at −80 °C for further experimental analyses.

#### Immunofluorescence, histopathological and ultrastructural examination

Immunofluorescence microscopic examination was performed to determine the effect of MG-infection on the number of CD8^+^ lymphocytes in the chicken thymus as described previously [29]. In brief, thymus samples were incubated with anti-CD8^+^ antibody (1:500, Bioss technology, Co. Ltd., Beijing, China) in blocking solution for 12 h at 4 °C after incubation with goat serum at room temperature. The slices were then incubated with CY3 (1:300, Service-bio Co. Ltd., Wuhan, China) labelled anti-rabbit IgG. After mounting with DAPI (Beyotime Biotechnology, Co, Ltd., Jiangsu, China), the sections were then examined under an inverted microscope (Nikon TE2000). Histopathological examination was performed as explained previously [30]. In brief, samples were processed in graded ethanol after fixing in 10% formalin overnight. After paraffin wax sectioning, the slices were stained with hematoxylin and eosin (Nanjing Chemical Reagent Factory, Nanjing, China) and examined under a light microscope (Nikon E100, Tokyo, Japan). Ultrastructural analysis was carried out as mentioned in a previous study [31]. Briefly, the samples were first fixed in 2.5% glutaraldehyde and rinsed twice for 15 min in 0.2 M PBS (pH = 7.2). Then, we fixed the specimens in 1% osmium tetroxide, dehydrated in graded ethanol and embedded in epoxy resin. The ultrathin sections were stained with lead citrate and uranyl acetate, and observed under a transmission electron microscope (GEM-1200ES, JEOL Ltd., Tokyo, Japan).

### Extraction of RNA and Real-time polymerase chain reaction (qRT-PCR)

Thymus tissue samples were lysed for 2 min at a low frequency of 65 Hz using an automatic tissue homogenizer (Shanghai Jingxin Industrial Development Co., Ltd.). Total RNA was extracted with TRIzol reagent (Invitrogen Inc., Carlsbad, CA, USA) as described previously [32]. RNA samples were then reverse-transcribed to first strand cDNA by a kit (Cat. # RR047A), purchased from Takara, Dalian, China. Genomic DNA was removed from the samples by treating them with gDNA eraser at 42 °C for 2 min. qRT-PCR was performed in a Roche LightCycler96 (Shanghai, China) instrument by using a kit according to the manufacturer’s instructions (Takara, Dalian, China, catalog no. RR820A). The β-actin gene was used as an internal standard and the data were analyzed by the Livak and Schmittgen method [33]. The list of primers used in qRT-PCR is shown in Table 1.

**Table 1:** **Primers used in qRT-PCR**

S. No.	Gene name	Primers (from 5′ to 3′)	Product Length
1	TLR2	Forward 5′-TCGCTCCAACACCTTCGCATTC-3′	181
		Reverse 5′-GATTGTCACCGTCGATCCTCAGC-3′	
2	NF-κB	Forward 5′-CACATGGTGGTGACCGCCAATAG-3′	194
		Reverse 5′-GTGCCATCGTATGTAGTGCTGTCC-3′	
3	Caspase-1	Forward 5′- GTGCTGCCGTGGAGACAACATAG-3′	179
		Reverse 5′- AGGAGACAGTATCAGGCGTGGAAG-3′	
4	NLRP3	Forward 5′- GCTCCTTGCGTGCTCTAAGACC-3′	150
		Reverse 5′- TTGTGCTTCCAGATGCCGTCAG-3′	
5	IL-10	Forward 5′- CAGCACCAGTCATCAGCAGAGC-3′	94
		Reverse 5′- GCAGGTGAAGAAGCGGTGACAG-3′	
6	IL-18	Forward 5′- AGATGATGAGCTGGAATGCGATGC-3′	97
		Reverse 5′- ATCTGGACGAACCACAAGCAACTG-3′	
7	PFK	Forward 5′-GTGAGAGTTGGCATAACGGAAGGC-3′	191
		Reverse 5′-CGCATCTGGTCAGCAATCTTCTCC-3′	
8	PK	Forward 5′-CTCAGCCAACTCTCCGTGATATGC-3′	175
		Reverse 5′-TCCACTGCTTCCAAGAACGATGAC-3′	
9	SDHB	Forward 5′-TGGACGGACTCTATGAGTGCATCC-3′	167
		Reverse 5′-TTGAAGTTGTGCCAGGCGTTCC-3′	
10	LDHB	Forward 5′-GCAGGTGTTCGTCAGCAAGAGG-3′	176
		Reverse 5′-GGCAGGCCACTCAACTTCCATG-3′	
11	LDHA	Forward 5′-TGCCTGTCTGGAGCGGAGTG-3′	116
		Reverse 5′-GTCCACCACCTGCTTGTGAACC-3′	
12	HK1	Forward 5′-TCATGGCTGTTGTGAACGATACCG-3′	132
		Reverse 5′-GGTCAATGTGCCGCATCTCCTC-3′	
13	HK2	Forward 5′-TGGAGGTGAAGCGGAGGATGAG-3′	177
		Reverse 5′-GCACCAGCAGCACACGGAAG-3′	
14	ACO2	Forward 5′-CCTGTGGACAAGCTGAGCATCG-3′	129
		Reverse 5′-CTGCGACTCGTTGAAGGTGTGG-3′	
15	MYD88	Forward 5′-AAGGTGTCGGAGGATGGTGGTC-3′ Reverse 5′-GGAATCAGCCGCTTGAGACGAG-3′	120
16	Beclin-1	Forward 5′-ACCGCAAGATTGTGGCTGAAGAC-3′	163
		Reverse 5′- TGAGCATAACGCATCTGGTTCTCC-3′	
17	mTOR	Forward 5′-AACCACTGCTCGCCACAATGC-3′	120
		Reverse 5′-CATAGGATCGCCACACGGATTAGC-3′	
18	TNF-α	Forward 5′-TGATCGTGACACGTCTCTGC-3′	88
		Reverse 5′- CAACCAGCTATGCACCCCAG-3′	
19	IL-6	Forward 5′-TTCACCGTGTGCGAGAACAGC-3′	80
		Reverse 5′- CAGCCGTCCTCCTCCGTCAC-3′	
20	IL-1β	Forward 5′-AGCAGCCTCAGCGAAGAGACC-3′	90
		Reverse 5′-GTCCACTGTGGTGTGCTCAGAATC-3′	
21	ULK-1	Forward 5′-AATCACAGACTCTGCTGGGC-3′	166
		Reverse 5′-AGTGTCCGCATAGTGTGAAGG-3′	
22	ATG5	Forward 5′- GGACGCATACCAACCTGCTT-3′	200
		Reverse 5′-TGCCATTTCAGTGGCGTACC-3′	
23	Dynein	Forward 5′-CGTTGCCAGCGTTACACCTATCC-3′	163
		Reverse 5′- GCCAGGACTGCCACCAACAC-3′	
24	β-actin	Forward 5′-CAACACAGTGCTGTCTGGTGGTAC-3′	199
		Reverse 5′-CTCCTGCTTGCTGATCCACATCTG-3′	

### Determination of ATPase activities

ATPase activities were examined in the thymus tissues using a kit (No. A016-2), purchased from Jiancheng Institute of Biotechnology (Nanjing, China). The activities of Na + -K + -ATPase, Mg ++-ATPase, Ca ++ATPase and Ca ++-Mg ++-ATPase were quantified at 660 nm by the level of inorganic phosphorus (Pi) produced as a result from the conversion of ATP to ADP. The inhibitors of all other types ATPase activities were added at the time of measurement of one type ATPase activity. The assays were performed in duplicate for each sample to avoid inter assay variation.

### Protein extraction and western blotting

Total proteins were extracted from thymus samples as mentioned in our previous study [34]. In brief, radioimmunoprecipitation assay (RIPA) and protease inhibitor phenylmethyl sulfonyl fluoride (PMSF) (Beyotime, China) were used to extract proteins, separated on SDS-PAGE (8–12%) and transferred to nitrocellulose membranes (Millipore, Bedford, MA, USA). The blots were then blocked and incubated with primary antibodies overnight at 4 °C. After washing with TBST solution, the membranes were incubated with secondary antibodies (goat anti-rabbit or anti-mouse horseradish peroxidase-conjugated IgG). After washing, the blots were visualized with enhanced chemiluminescence (ECL, Biosharp Life Sciences, China) reagent and analyzed by Image J software (Version 1.42, National Institutes of Health, USA).

### Statistical analysis

All the experiments were performed in triplicates unless otherwise mentioned (*n* = 3). The results were expressed as mean ± standard deviation (SD). Statistical significance was determined at a value of *p* < 0.05 by using statistical package for social sciences software (SPSS, version 21.0) through *t* test and the bar graphs were made by GraphPad Prism (San Diego, California version 6.01).

## Results

### MG-infection reduced the number of CD8^+^ lymphocytes in thymus tissues

**Figure 1:**
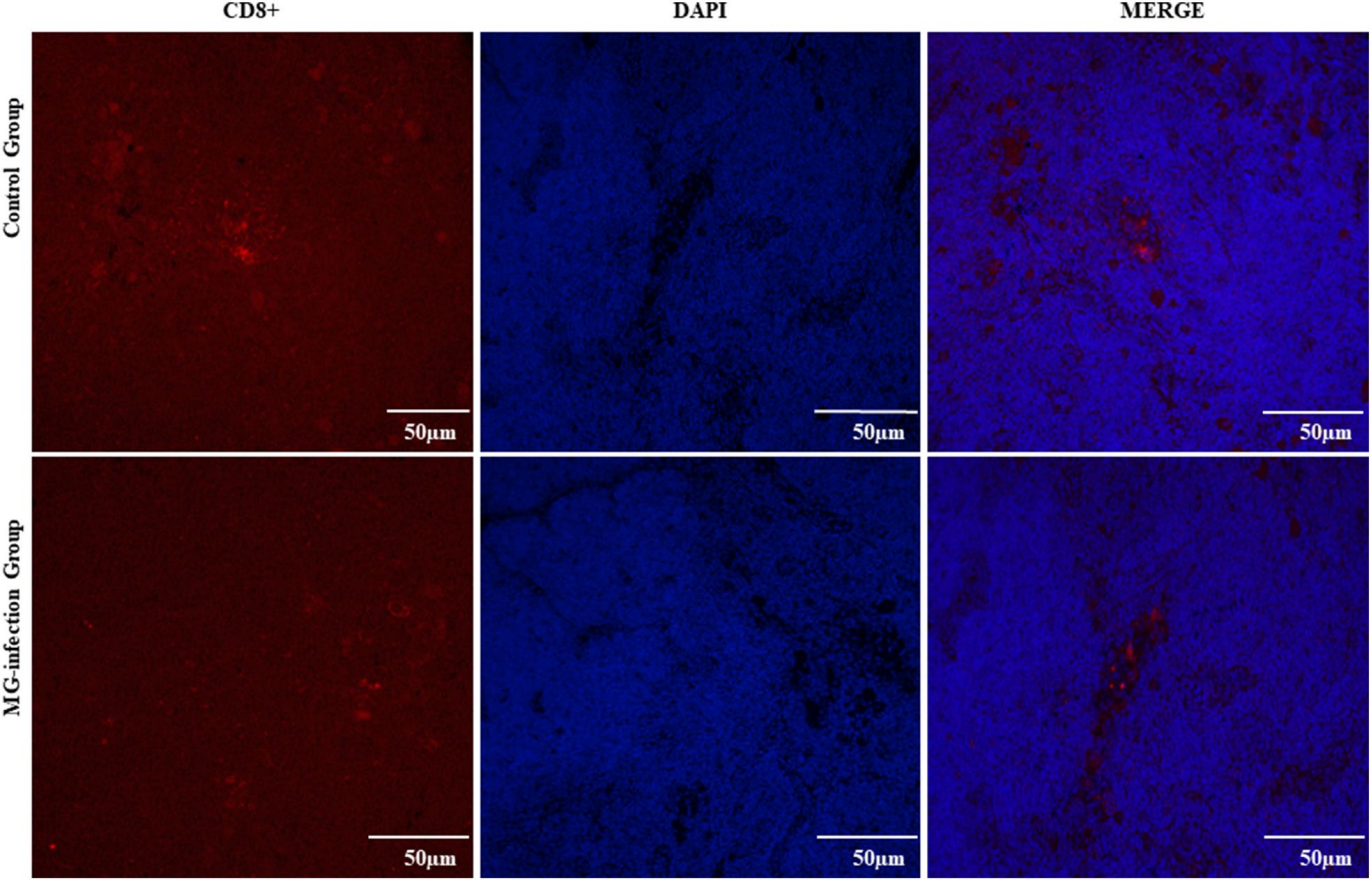
**The effect of MG-infection on the number of CD8**^**+**^**T-lymphocytes were estimated by using immunofluorescence microscopy**. The photomicrographs were taken at 400 × magnification (*n* = 3). Groups are represented as control group and MG-infection group. The immunofluorescence photomicrographs are represented as CD8^+^, DAPI and Merge in each group.

We examined the number of CD8^+^ T-lymphocytes in thymus tissues to determine whether MG-infection induced depletion of CD8^+^ T-lymphocytes in chicken thymus. MG-infection caused depletion of CD8 + cells and resulted in a significant decrease in the number of CD8^+^ lymphocytes in the thymus tissues compared to the control group (as shown in Figure 1). The reduction in the number of CD8^+^ cells in the thymus could be the possible reason of immune dysregulation during MG-infection.

### MG-infection impaired thymus ultrastructural and histological morphology

**Figure 2:**
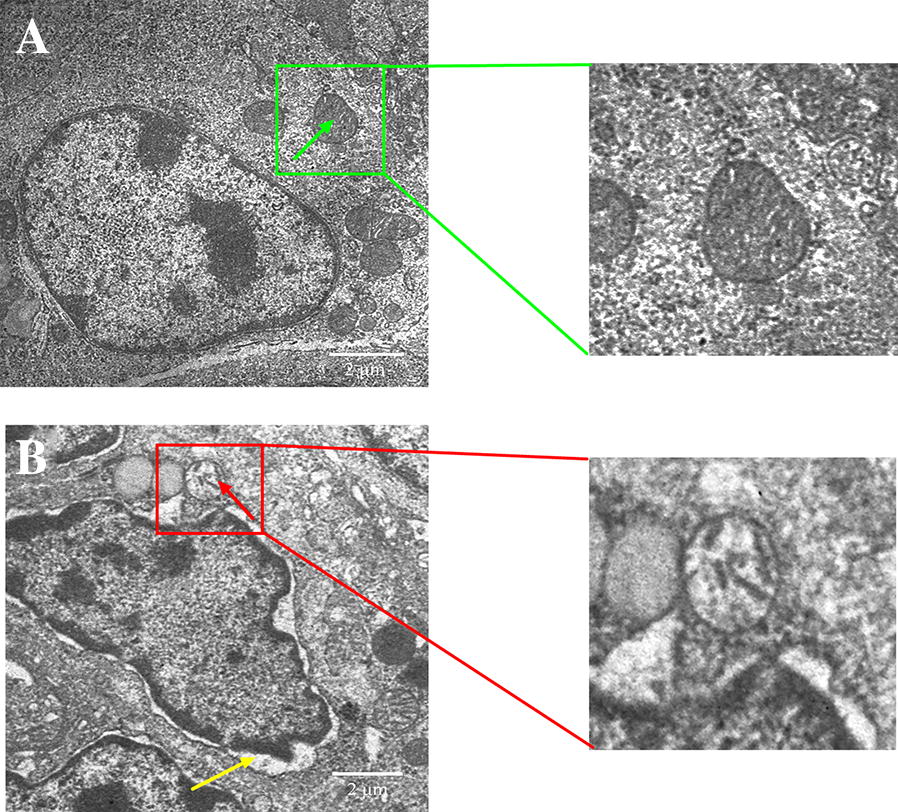
**Ultrastructural analysis showing the effect of MG-infection on chicken thymus**. Ultrastructural analysis (Figure 3) was performed at day 7 post-infection. Experimental groups are represented as (**A**) control group and (**B**) MG-infection group (*n* = 3). It is clear from the ultrastructural photomicrographs (zooming area) that the mitochondria (green arrow) are intact and clearly visible in the control group. In contrast, mitochondrial swelling (red arrow), increased intercellular space, nuclear lysis, and cell membrane deformation (yellow arrow) were present in the MG-infection group.

**Figure 3:**
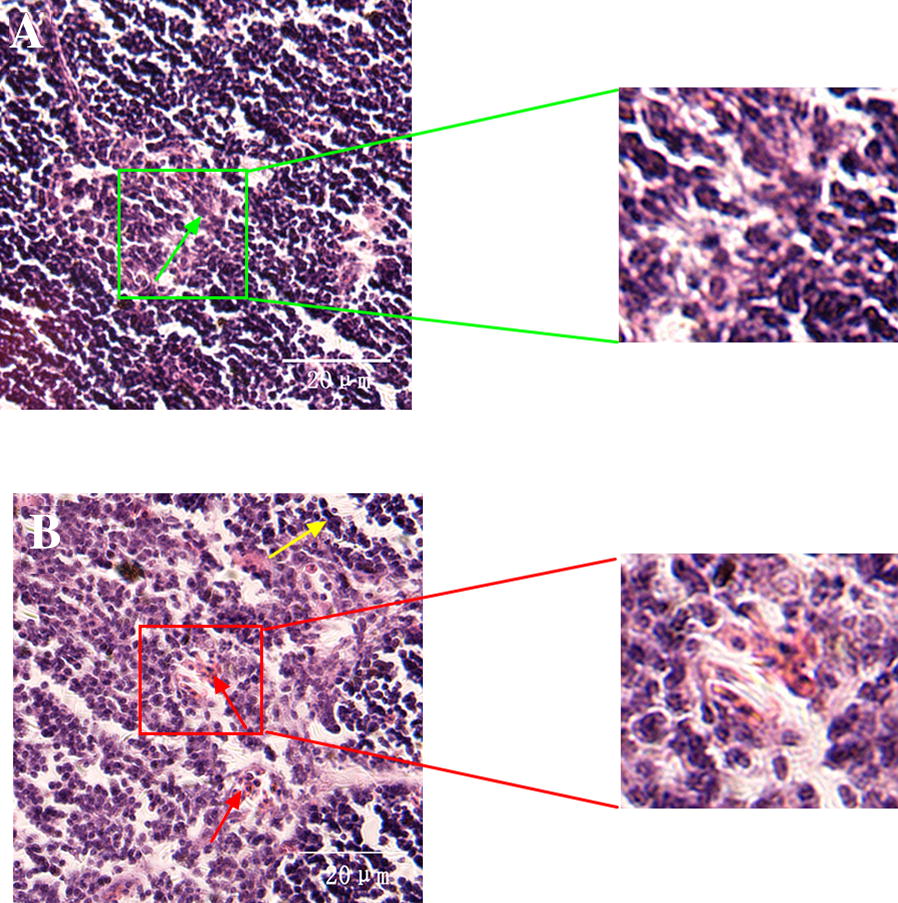
**Histological examination showing the effect of MG-infection on morphology of thymus tissues**. The stained Sects. (40 × magnification) are represented as (**A**) control group (**B**) and MG-infection group (*n* = 3). The green arrow shows normal intact structure of thymus tissues in the control group, while yellow arrow shows necrotic debris and red arrow shows that thymus tissues lose their compact arrangement with increased inflammatory cells infiltrates.

The ultrastructural changes in thymus tissues are shown in Figure 2. Ultrastructural analysis revealed typical features of apoptosis such as swollen mitochondria, cell shrinkage, membrane deformation and chromatin condensation in the MG-infection group (Figure 2B) compared to the control group (Figure 2A). Moreover, the nuclear membrane disappeared and lost its structural integrity in the MG-infection group. However, thymus tissues from the control group showed no significant abnormal morphological changes compared to the MG-infection group (Figure 2A). The results of histological examination are shown in Figure  3. Regular morphology with intact tissue structures and a clear boundary was observed between the thymus cortex and medulla in the control group (Figure 3A). In contrast, MG-infection (Figure 3B) caused severe structural alterations, triggered inflammatory cell infiltration in the medulla region and increased nuclear debris in the thymus tissues. These signs correlate with MG-induced inflammatory response and immune damage in chicken thymus.

### MG-infection induced NLRP3 activation via TLR-2/MyD88/NF-κB pathway

**Figure 4:**
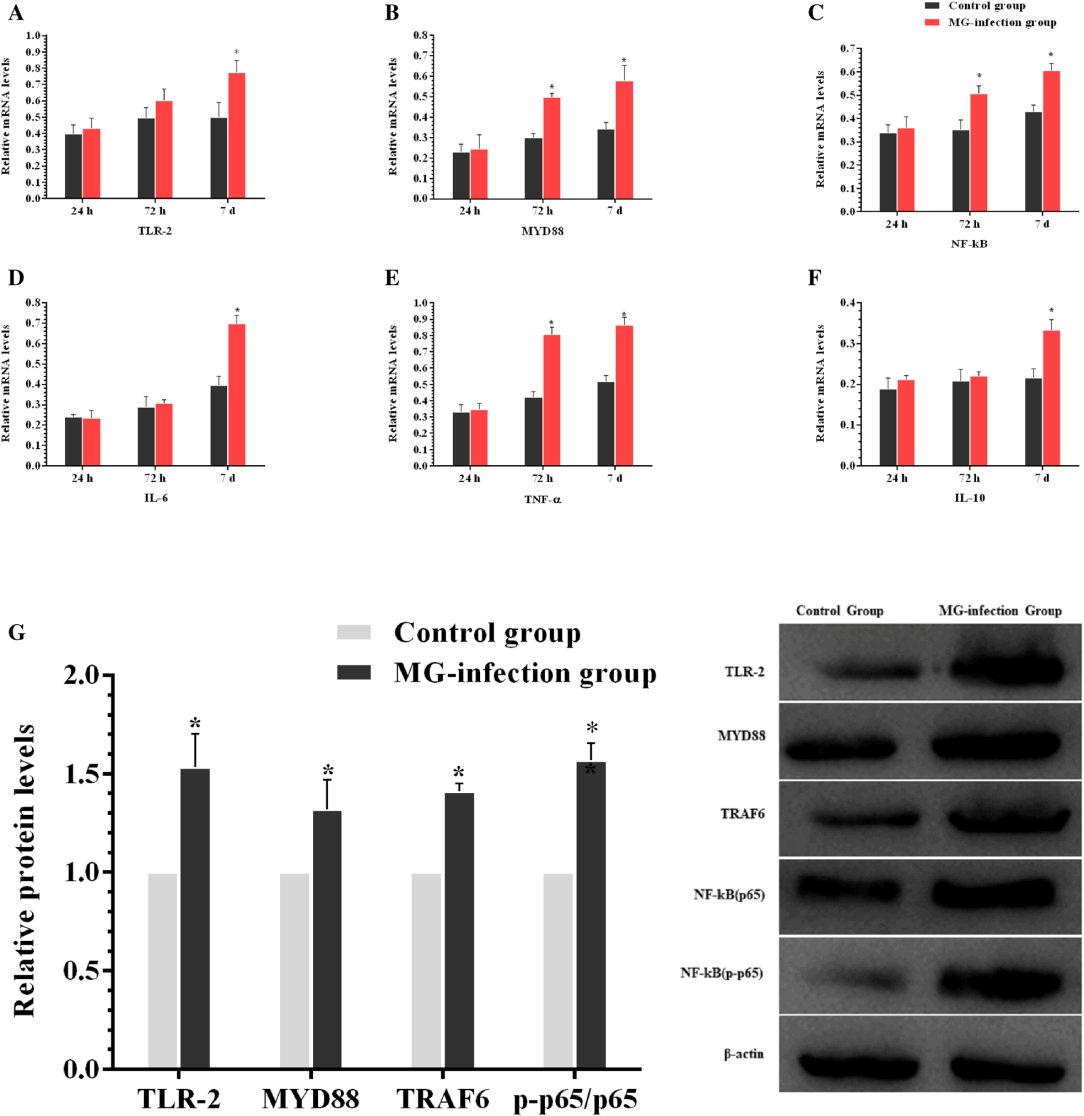
**MG-infection induced TLR-2/MyD88/NF-κB pathway**. Panels **A–F** represent mRNA levels of inflammation-related genes at the three time points and panel **G** represents protein expression level. Experimental groups are represented as control group and MG-infection group. Bar graphs represent mean results ± SD (*n* = 3). *P < 0.05 represents statistically significant difference compared to the control group.

**Figure 5:**
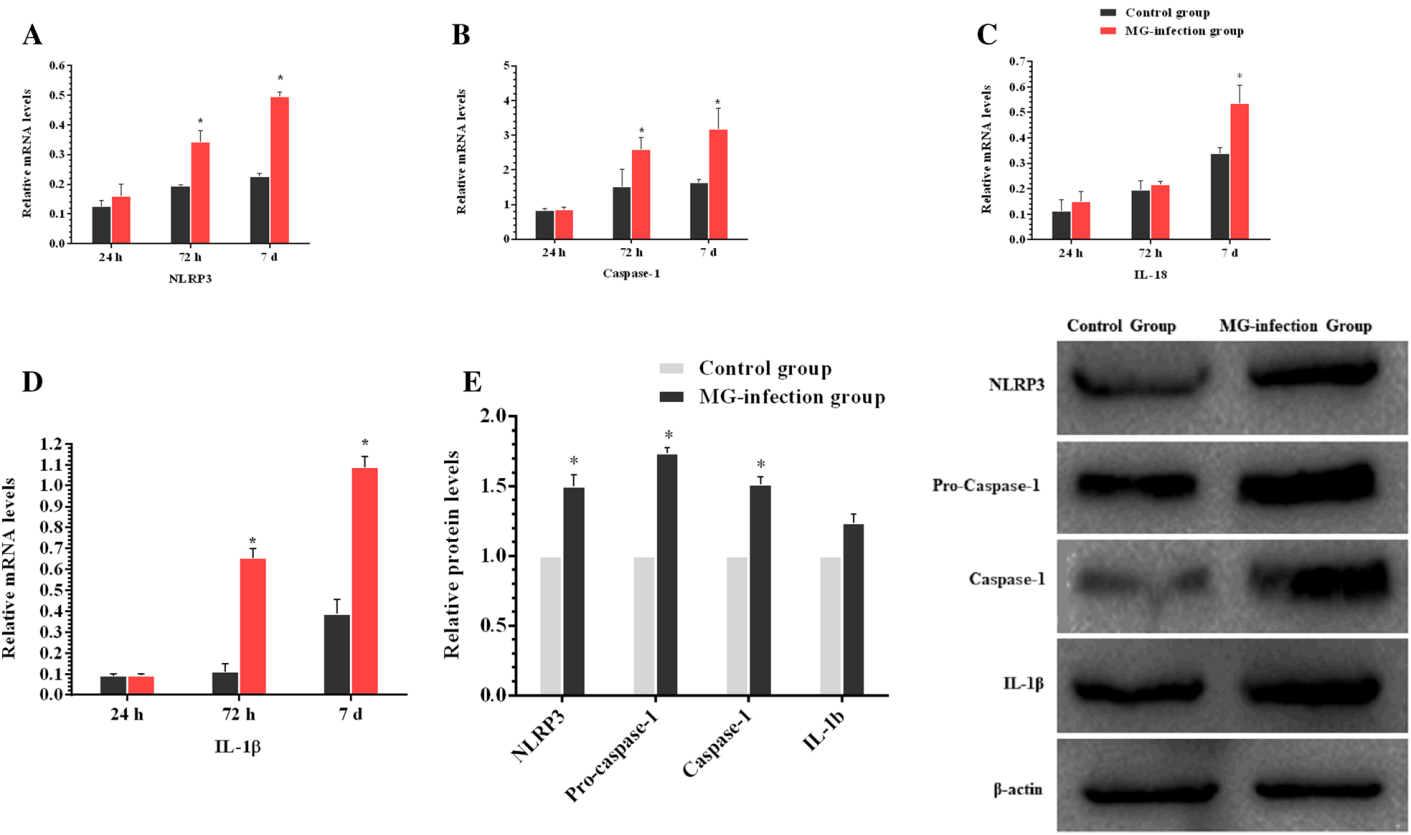
**MG-infection activated NLRP3 inflammasome in chicken thymus**. Panels **A-D** represent mRNA levels at the three time points and panel **E** shows protein levels of the two experimental groups at day 7. Experimental groups are represented as control group and MG-infection group. Bar graphs represent mean results ± SD (*n* = 3). *P < 0.05 represents statistically significant difference compared to the control group.

TLR-2/MyD88/NF-κB pathway plays a crucial role in inflammatory responses. Therefore, we determined the expression of TLR-2/MyD88/NF-κB pathway in chicken thymus (as shown in Figure 4). It has been noted that MG-infection significantly enhanced the mRNA expression of TLR-2, MYD88, NF-κB, TNF-α, IL-6 and IL-10 gene compared to the control group at all the three time points. The increase in the mRNA expression of MYD88, NF-κB and TNF-α was significant at day 3 and day 7 compared to the control group. TLR-2, MYD88, TRAF6 and phosphorylated NF-κB protein levels were also significantly upregulated at day 7 in the MG-infection group compared to the control group. Furthermore, we examined the mRNA and protein expression levels of NLRP3 inflammasome-related genes in thymus tissues (Figure 5). MG-infection increased mRNA levels of NLRP3, caspase-1, IL-18 and IL-1β gene in thymus tissues at the three time points. The increase in mRNA expression levels of NLRP3, caspase-1 and IL-1β was statistically significant at day 3 and day 7. For IL-18, the difference was only significant at day 7. In addition, the protein expression levels of NLRP3 (*p* < 0.05), caspase-1 (*p* < 0.05) and IL-1β (*P *> 0.05) increased at day 7 in the MG-infection group compared to the control group. These results suggested that MG-infection-mediated inflammatory response involved NLRP3 activation through NF-κB pathway in the chicken thymus.

### Effect of MG-infection on ATPase activities in thymus tissues

**Figure 6:**
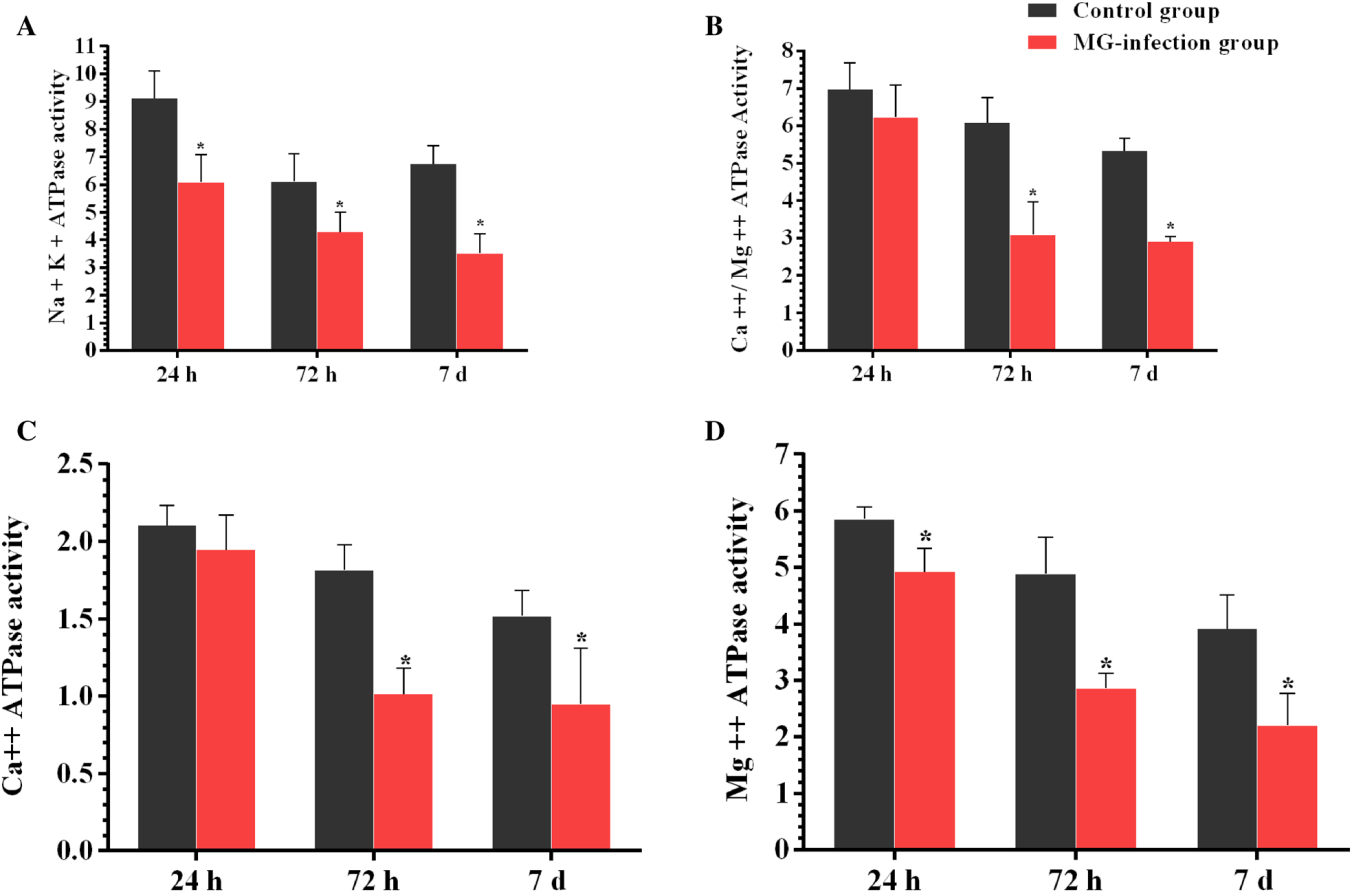
**MG-infection suppressed ATPase activities in chicken thymus**. Panels **A** –**D** show the effect of MG-infection on Mg ++-ATPase, Ca ++-Mg ++-ATPase, Ca ++ATPase and Na + -K + -ATPase activities, assessed at the three time points. Experimental groups are represented as control group and MG-infection group. Bar graphs represent mean results ± SD (*n* = 3). *P < 0.05 represents statistically significant difference compared to the control group.

The effects of MG on ATPase activities are represented in Figure 6. MG-infection suppressed Mg ++-ATPase, Ca ++-Mg ++-ATPase, Ca ++ATPase and Na + -K + -ATPase activities at the three time points in the chicken thymus. The decrease in Mg ++-ATPase and Na + -K + -ATPase activities were found statistically significant (*p* < 0.05) at the three time points. Ca ++-Mg ++-ATPase and Ca ++ATPase decreased activity were significant only at day 3 and day 7 in the MG-infection group compared to the control group.

### MG-infection modulated autophagy and energy metabolism in chicken thymus

**Figure 7:**
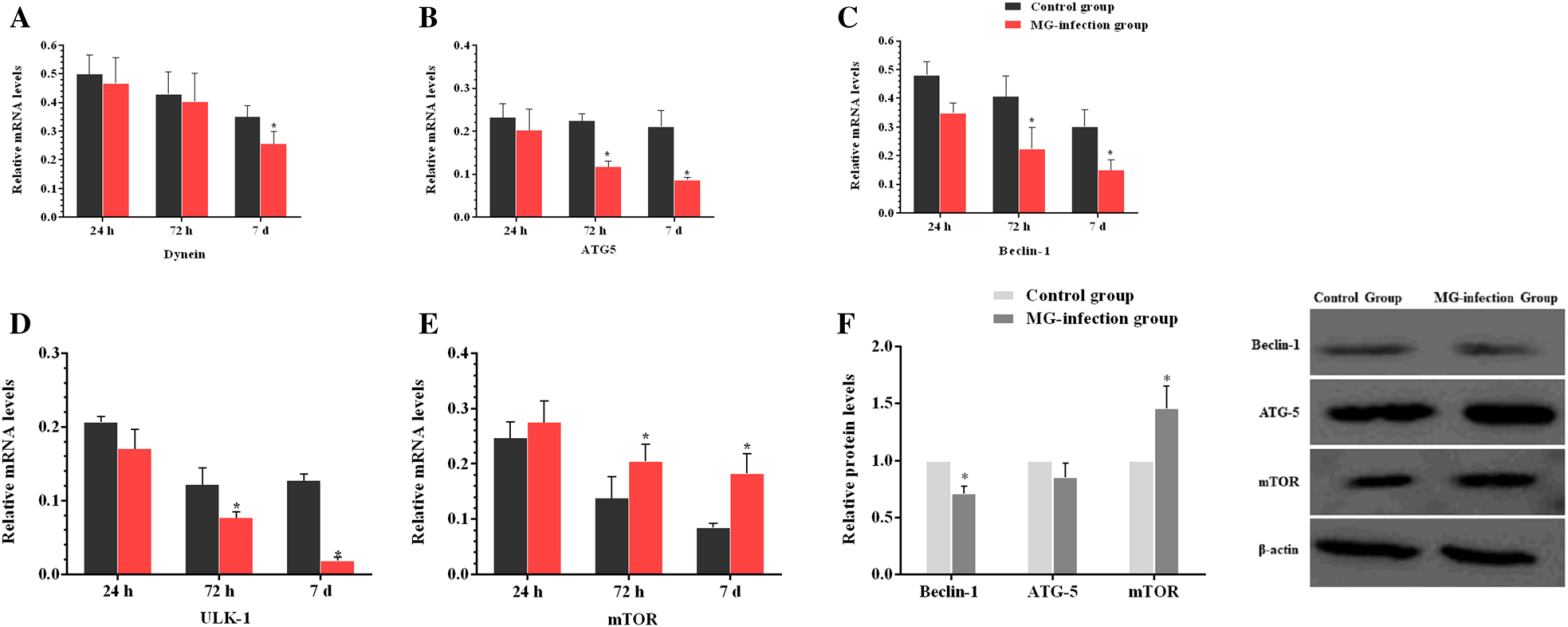
**MG-infection modulated autophagy in chicken thymus tissues**. Panels **A–E** display mRNA expression levels of autophagy-related genes at the three time points and panel **F** shows protein expression level at day 7. Experimental groups are represented as control group and MG-infection group. Bar graphs represent mean results ± SD (*n* = 3). *P < 0.05 represents statistically significant difference compared to the control group.

**Figure 8:**
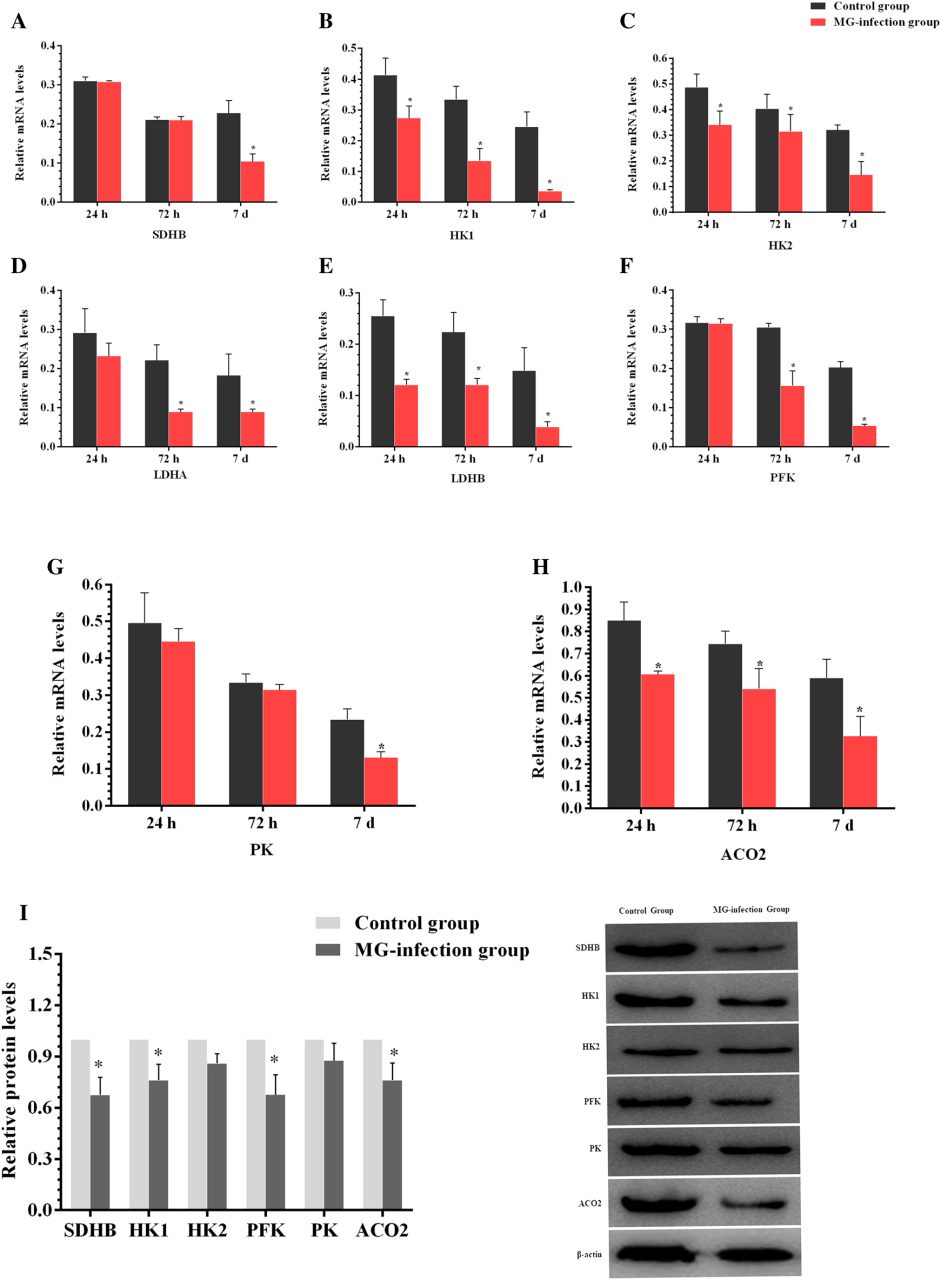
**Effect of MG-infection on energy metabolism-related genes in chicken thymus**. Panels **A–H** represent mRNA expression levels of energy metabolism-related genes at the three time points and panel **I** represents protein expression levels at day 7. Experimental groups are represented as control group and MG-infection group. Bar graphs represent mean results ± SD (*n* = 3). *P < 0.05 represents statistically significant difference compared to the control group.

We examined the expression of autophagy-related genes both at mRNA and protein level in order to determine the effect of MG-infection on autophagy. The results are shown in Figure 7. MG-infection decreased Dynein, ATG-5, Beclin-1 and ULK-1 gene mRNA expression compared to the control group at the three assessed time points. The decrease in ATG-5, Beclin-1 and ULK-1 gene mRNA expression were statistically significant (*p* < 0.05) at day 3 and day 7 compared to the control group. In contrast, the mRNA expression of mTOR was significantly (*p* < 0.05) enhanced at day 3 and day 7 in the MG-infection group compared to the control group. Moreover, the protein expression levels of Beclin-1 (*p* < 0.05) and ATG-5 (*p* > 0.05) gene were reduced in chicken thymus, except mTOR level, which was significantly (*p* < 0.05) enhanced in the MG-infection group compared to the control group. In addition, we examined the expression level of energy metabolism-related genes in thymus tissues (Figures 8A–I). Compared to the control group, MG-infection caused a significant (*p* < 0.05) reduction in HK1, HK2, LDHB and ACO2 gene mRNA expression at the three time points. However, the decrease in LDHA and PFK gene mRNA expression were significant at day 3 and day 7, except SDHB and PK, where the mRNA expression was significant (*p* < 0.05) only at day 7. The protein expression results (Figure 8I) showed similar trends. The protein level of SDHB (*p* < 0.05), HK1 (*p *< 0.05), HK2 (*p *< 0.05), PFK (*p *< 0.05), PK (*p *> 0.05) and ACO2 (*p *< 0.05) were decreased in the MG-infection group compared to the control group.

## Discussion

The thymus plays a central role in immune responses as it is a place for T-cell development, maturation, differentiation and recruitment [35]. CD4^+^CD8^+^ double-positive cells constitute 80% of the whole thymocytes population. These CD4^+^CD8^+^ double-positive cells undergo complex recruitment and programmed selection process and become single positive thymocytes. Previous studies reported that this selection process was affected by foreign invading pathogenic infections [36, 37]. In the present study, fluorescence microscopy results showed that the number of CD8^+^ T-lymphocytes were significantly decreased in the MG-infection group compared to the control group. In addition, electron microscopy and morphological analysis revealed structural alterations, increased inflammatory cells infiltrations, mitochondrial and DNA damage in the thymus of MG-infected chickens. These findings indicated that MG-infection induced structural and immune damage through the depletion of T-lymphocytes. This depletion might be due to the MG-mediated induction of apoptosis in thymic tissues. Another study reported mild to moderate congestion, microscopic lesions and lymphocyte depletion in the thymus of chickens infected with MG [38]. These results are in line and further confirmed that MG-induced apoptosis and oxidative stress and damage the structural integrity of chicken thymus tissues [26]. Previously, MG-induced inflammatory responses in the lungs of chickens had been well demonstrated [8, 31]. However, thymus plays a vital role in protection against invading pathogens and regulating immune responses. Therefore, additional studies are needed to investigate the effect of MG on chicken thymus.

**Figure 9:**
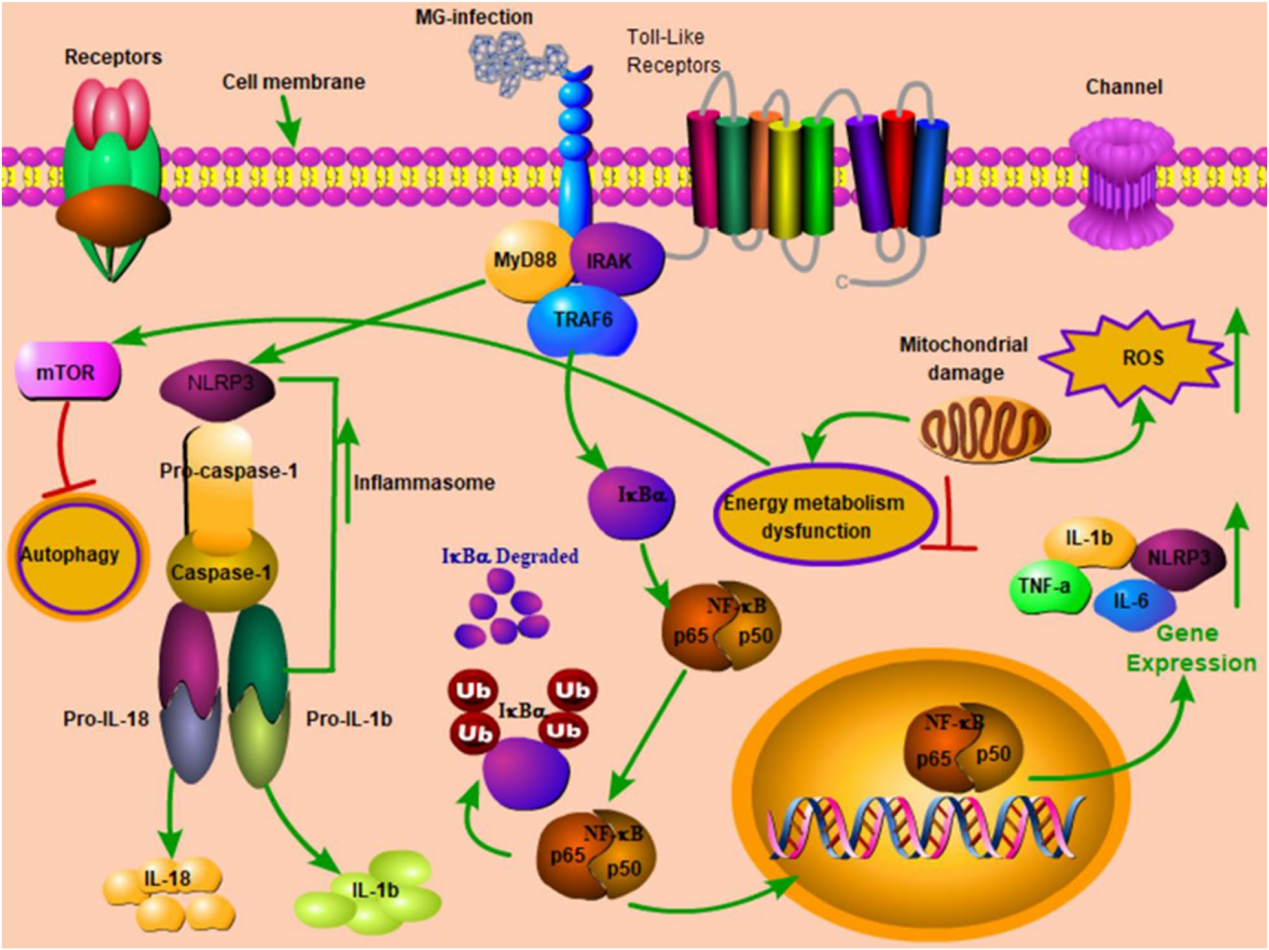
**Schematic diagram showing the effect of MG-infection on chicken thymus tissues**. MG-infection activated NLRP3 inflammasome involving TLR-2/MyD88/NF-κB pathway, decreased the level of autophagy and induced energy metabolism dysfunction. Red arrows show inhibition/downregulation and green arrows show upregulation or increased expression and connection between signaling molecules.

TLRs are transmembrane receptors, which come in direct contact with pathogens, mainly expressed on immune and epithelial cells that recognize PAMPs [39]. Cumulative evidence showed that TLRs in connection with NLRs activated inflammasome such as NLRP3 and induced the secretion of IL-1β [40]. Aberrant and continuous inflammasome activation is associated with immune disorders and autoinflammatory diseases [41, 42], including Muckle-Wells syndrome (MWS), familial cold autoinflammatory syndrome (FCAS) and neonatal-onset multisystem inflammatory disease (NOMID) [43]. Previous study reported that NLRP3 inflammasome is a critical regulator during *Mycoplasma pneumoniae* infection [44, 45]. However, the mechanism of NLRP3 inflammasome activation is still not reported in chicken thymus. Our data showed that TLR2-MYD88-NF-κB signaling pathway was significantly upregulated in the thymus of MG-infected chickens. In addition, the mRNA and protein expression levels of NLRP3, Caspase-1 and IL-1β were significantly enhanced in the MG-infection group. These results suggested that TLR2-MYD88-NF-κB signaling pathway was involved in inflammasome activation in chicken thymus. From these results, it could be speculated that NLRP3 inflammasome activation is possibly involved in immune dysregulation in chicken thymus during MG-infection. Furthermore, researchers reported that TLRs also regulate macroautophagy (also known as autophagy) [46]. Autophagy provides cytoprotection for homeostatic control, clearance of cytosol invading microbes and helps in the restoration of nutrient supply during starvation [47]. It is of prime importance to study the metabolic host responses to mycoplasmal infections to better understand bacterial pathogenesis. Previously, researchers mainly focused on pathogen triggered immune responses, endosomal vesicle formation, apoptosis, autophagy, host cell survival and inflammation [48, 49], and linked energy metabolism, inflammatory responses, cell transformation to nitrogen by signaling pathways and/or shared transcriptional regulators [50–53], while, the effect of bacterial infections on host metabolic responses is rarely reported [54]. In the present study, MG-infection caused a significant reduction in the expression of autophagy and energy metabolism-related genes both at mRNA and protein level in the thymus of MG-infected chickens. In general, the decrease in energy metabolism correlates with the increase in inflammatory responses and inflammasome activation reduced autophagy, which results in thymus tissue damage during MG-infection. The present study provides evidence that TLR-2/MyD88/NF-κB signaling pathway and NLRP3 inflammasome activation could be involved in MG-induced immune dysregulation in chicken thymus as shown in the schematic diagram (Figure 9). However, further molecular studies are needed to scrutinize the detail molecular mechanisms between inflammasome activation and depletion of energy at cellular and molecular level in chicken lymphoid organs.

In conclusion, MG-infection caused reduction in the number of CD8^+^ lymphocytes and suppressed ATPase activities. In addition, MG-infection impaired the structural integrity of thymus tissues, weakened energy metabolism and reduced the level of autophagy. Taken together, the study provides promising therapeutic targets for future pharmacological studies to control MG-induced immune dysregulation and inflammatory responses in chickens.

## Data Availability

The datasets analyzed during the current study are available from the corresponding author on reasonable request.
